# Hydrogen in the Earth core inferred from neutron imaging and diffraction

**DOI:** 10.1038/s41598-026-49969-z

**Published:** 2026-05-11

**Authors:** Naoki Takahashi, Tatsuya Sakamaki, Takanori Hattori, Ken-ichi Funakoshi, Hiroshi Arima-Osonoi, Asami Sano-Furukawa, Jun Abe, Akio Suzuki

**Affiliations:** 1 Department of Earth Science, Graduate School of Science, Tohoku University, 6-3 Aoba, Aramaki, Aoba-ku, Sendai, 980-8578 Japan; 2 J-PARC Center, Japan Atomic Energy Agency, Tokai, Naka, 319- 1195 Ibaraki Japan; 3 Neutron Science and Technology Center, Comprehensive Research Organization for Science and Society, Tokai, Naka, 319-1106 Ibaraki Japan; 4 Institute for Integrated Radiation and Nuclear Science, Kyoto University, Osaka, 590-0494 Japan

**Keywords:** Core processes, Geochemistry, Core processes

## Abstract

The density of the Earth’s core is lower than that of pure iron; this is considered to be caused by the presence of light elements in the core. Hydrogen is one of the most important light elements in the Earth’s core because of its high cosmochemical abundance and its nature as a siderophile element under high pressure. Thus, the hydrogen content in liquid iron under high pressure is required to constrain the chemical composition of the Earth’s core. However, the hydrogen content has been estimated based on the observation of quench products; there are no examples of hydrogen content being determined in the liquid state. Here, we performed high-pressure and high-temperature neutron diffraction and imaging experiments in situ to determine the hydrogen content in liquid iron. We observed that liquid iron contains 0.17(3) wt% H at 3.4 GPa and 1400 K, indicating that liquid iron is hydrogenated in the magma ocean during core formation. For the hydrogen content in the liquid iron at the base of the magma ocean, we estimated that the outer and inner cores contain 0.60–0.72 and 0.30–0.44 wt% H, corresponding to 70–85 and 1.9–2.7 times the mass of hydrogen in the ocean, respectively. This suggests that hydrogen can contribute more than half of the density deficit in the outer core. For the magma ocean equilibrating with the hydrogen-rich primary atmosphere, the study findings show that liquid iron plays a crucial role in transporting a large amount of hydrogen into the core.

## Introduction

The Earth’s core primarily comprises iron, nickel (5 wt%), and certain light elements, such as silicon, sulfur, oxygen, carbon, and hydrogen^1–3^. Owing to the light elements in the Earth’s core, the density of the core observed geophysically is lower than the density of pure iron^1–3^. In particular, hydrogen, the lightest and most abundant element in the solar nebula, is an important element in the Earth’s core because its solubility in iron significantly increases under high pressure^4^, and hydrogen becomes increasingly siderophile under pressure^5–7^. However, hydrogen rapidly escapes from iron because iron hydride (FeH_*x*_) is only stable under high pressures and decomposes into α-Fe with a body-centered-cubic structure and molecular H_2_ under ambient conditions^8,9^. Thus, owing to the aforementioned experimental difficulty, hydrogen has been less investigated than other light elements. In addition, although the composition of solid FeH_*x*_/FeD_*x*_ is determined via in situ X-ray diffraction (XRD)^4,7,10–19^ or neutron diffraction^9,20–23^, the hydrogen content in liquid FeH_*x*_ is not easily measured in situ. Experimental studies on liquid FeH_*x*_ have determined the hydrogen content using indirect methods. Okuchi^24^ estimated the hydrogen content in liquid FeH_*x*_ using a rapid-decompression technique to trap the hydrogen as numerous bubbles before it escaped from liquid iron. In addition, in situ XRD studies have been performed to determine the hydrogen content in liquid FeH_*x*_ assuming that it is equivalent to the content measured in solid quench products^7,17–19^ since hydrogen escapes from α-Fe during decompression to 1 atm^8,9^. Thus, a method for determining the hydrogen content in liquid FeH_*x*_ in situ is required.

Here, we performed in situ neutron diffraction and imaging experiments for FeH_*x*_ at 3 GPa and 300–1400 K to determine the hydrogen content in liquid FeH_*x*_. Using the neutron diffraction data, we precisely determined the hydrogen content in an iron lattice by Rietveld refinement. Neutron radiography images were used to calculate the absorption coefficients. Thereafter, we determined the hydrogen content in liquid FeH_*x*_ using the absorption coefficient of liquid FeH_*x*_ and the calibration curve obtained from the mass absorption coefficients and hydrogen contents in solid phases. Finally, we estimated the hydrogen content in the Earth’s core, suggesting that hydrogen can cause more than half of the density deficit in the outer core.

## Results of neutron diffraction experiments

**Fig. 1:**
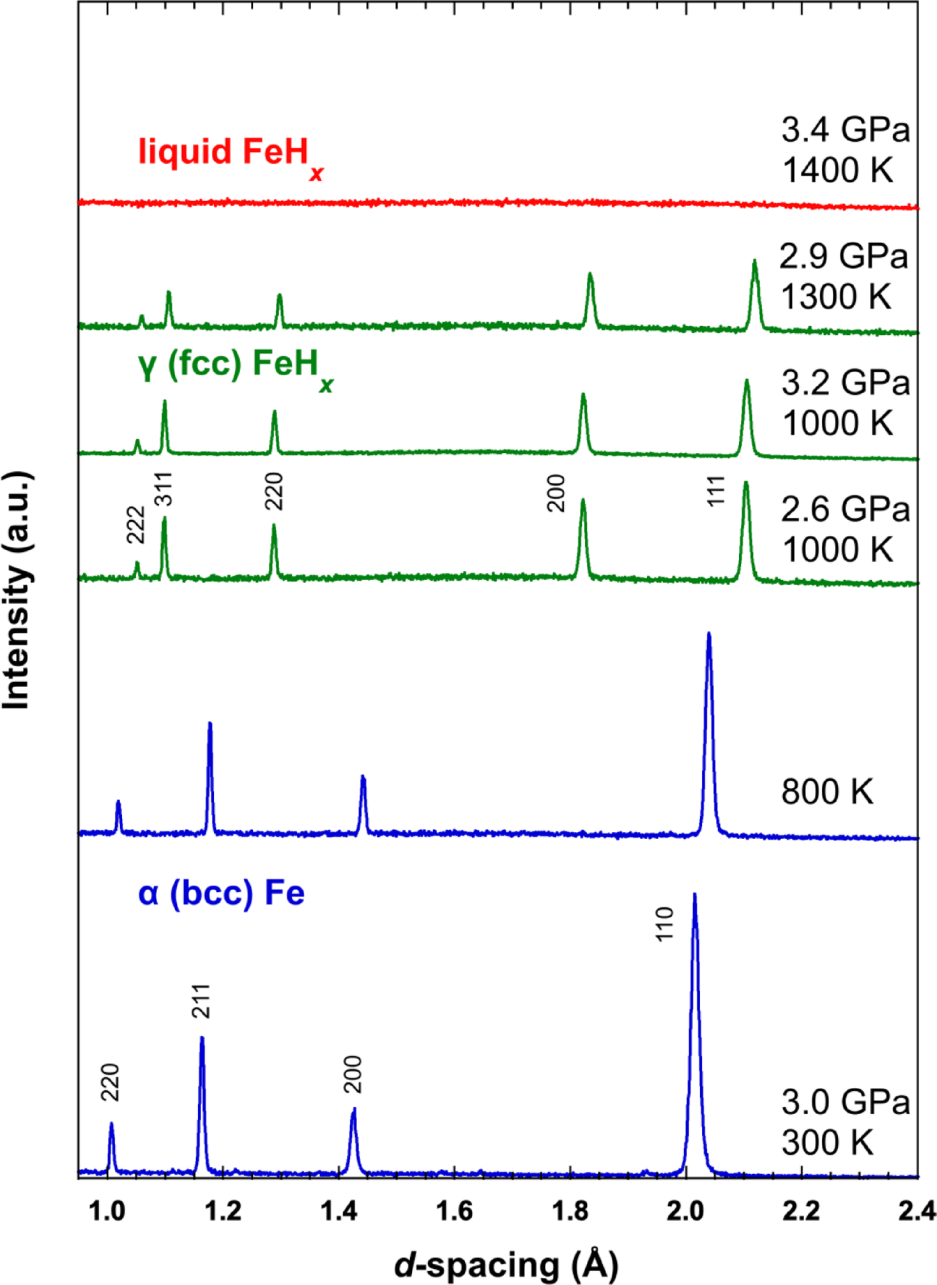
Sequential neutron diffraction profiles recorded during heating at approximately 3 GPa. The blue, light-green, and red lines represent the neutron diffraction profiles of α-Fe, γ-FeH_*x*_, and liquid FeH_*x*_, respectively.

We performed neutron diffraction and imaging experiments in situ at approximately 3 GPa and 300–1400 K using a six-axis multi-anvil apparatus (ATSUHIME)^25^ installed in the PLANET beamline^26^ at the Materials and Life Science Experimental Facility (MLF) in J-PARC. Figure 1 shows the diffraction profiles recorded during heating at approximately 3 GPa. Here, the phase transition from α-Fe to γ-FeH_*x*_ (a face-centered cubic (fcc) structure) occurred within the range of 800–900 K, consistent with the phase boundary reported by Kakizawa et al.^27^. The diffraction peaks of γ-FeH_*x*_ fully disappeared at 3.4 GPa and 1400 K. Thus, the sample was considered fully melted at 3.4 GPa and 1400 K.

Here, the hydrogen content in α-Fe was negligible since the H solubility in α-Fe is extremely low^12,28,29^, and Rietveld refinement was conducted for only γ-FeH_*x*_. The γ-Fe lattice has two interstitial sites for holding hydrogen atoms: octahedral (O) and tetrahedral (T) sites. In transition metals with an fcc lattice, the dissolved H atoms preferentially occupy the O-site because of its larger free space compared with that of the T-site. Rietveld refinement was performed for the three structural models of γ-FeH_*x*_ reported by Machida et al.^20^ and Ikuta et al.^22^: (A) the γ-Fe lattice without H atoms, (B) γ-Fe lattice with H atoms in only the O-site, and (C) γ-Fe lattice with hydrogen atoms occupying the O- and T-sites. Thus, the structural parameters, including the site occupancies of H in the O- and T-sites ($\:{g}_{\mathrm{H}\left(\mathrm{O}\right)}$ and $\:{g}_{\mathrm{H}\left(\mathrm{T}\right)}$), were refined (see “Methods”).

**Table 1:**
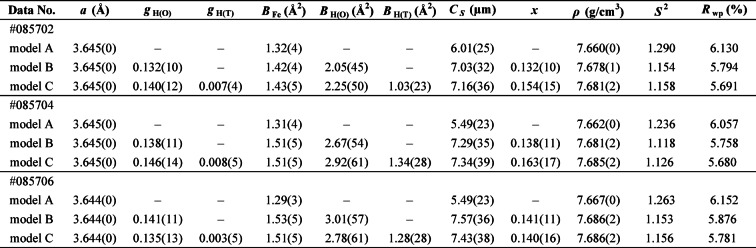
Refined structural parameters using models A–C

Rietveld refinement results for γ-FeH_x_ measured for 1 h at 3.2 GPa and 1000 K. The values in parentheses represent the error of each parameter. *a*: unit cell parameter for γ-FeH_x_; *g*_H(O)_ and *g*_H(T)_: site occupancy of H atom in the octahedral and tetrahedral sites, respectively; *B*_Fe_, *B*_H(O),_ and *B*_H(T)_: isotropic atomic displacement parameters of the Fe atom, H atom in the octahedral site, and H atom in the tetrahedral site, respectively; C_S_: crystallite size for extinction correction;*x*: hydrogen content in γ-FeH_x_ [= *g*_H(O)_ + 2 *g*_H(T)_]; *ρ*: density; *S*^2^: goodness-of-fit; *R*_wp_: reliability factor.

**Fig. 2:**
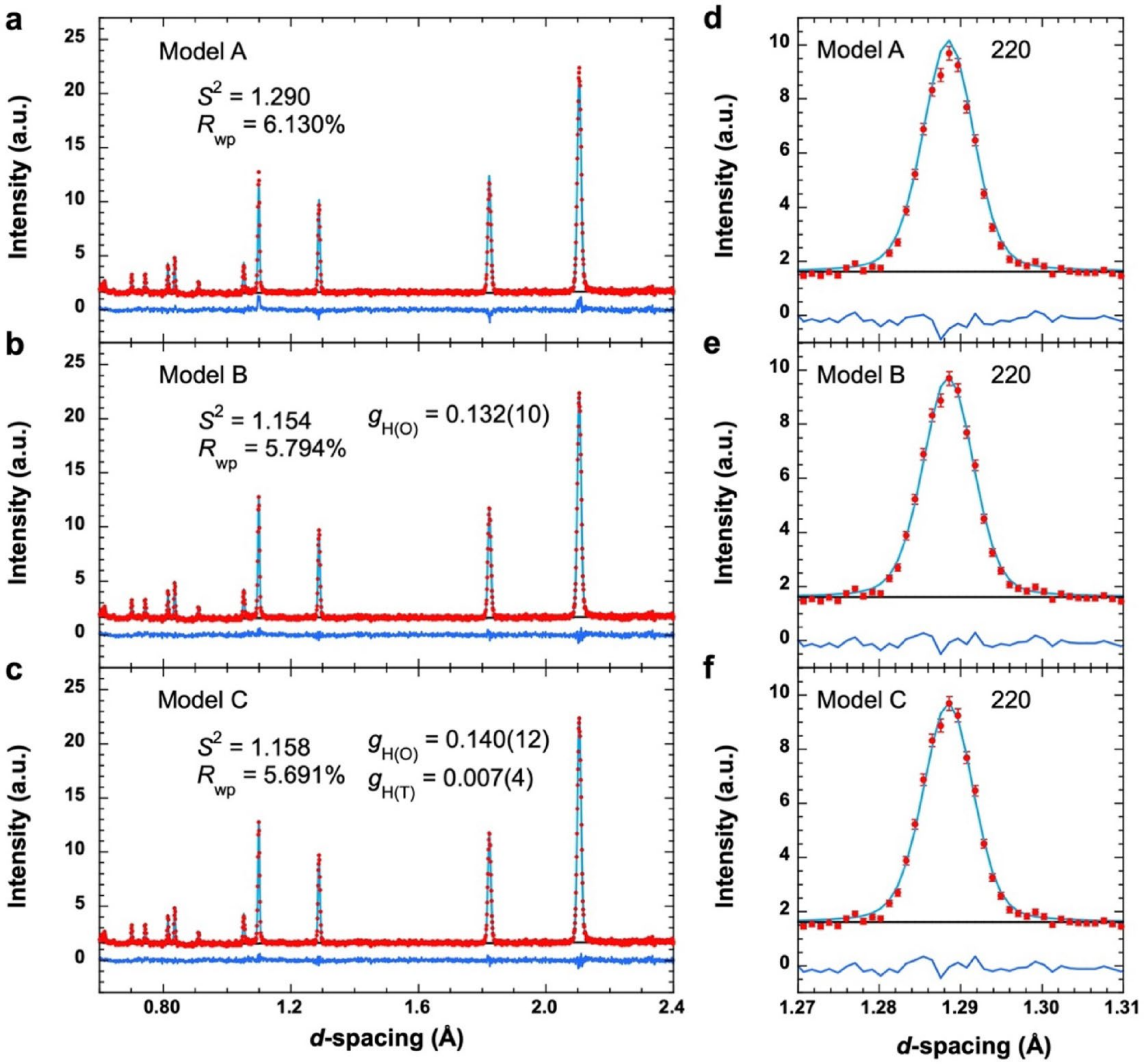
Typical experimental diffraction profiles and fitting results of Rietveld refinement for γ-FeH_*x*_ (Data #085702). Refined profiles calculated using (**a**) models A, (**b**) B, and (**c**) C. The red circles represent the experimentally measured neutron diffraction profile. The light-blue line represents the profiles calculated from Rietveld refinement. The blue line represents the difference between the experimental and calculated profiles. The black line represents the background of the experimentally obtained neutron diffraction profile. (**d**–**f**) Enlarged 220 diffraction peak profiles, in which the experimental errors are represented as vertical bars. Better refinements were achieved using hydrogenation models B and C rather than non-hydrogenation model A, and no clear difference was observed between models B and C.

Figure 2 shows the neutron diffraction data obtained at 3.2 GPa and 1000 K (Data #085702) with Rietveld fitting. Extended Data Table 1 presents the results of the refined structural parameters, including the results of Data #085704 and #085706, which were measured for 1 h at 3.2 GPa and 1000 K. As shown in Fig. 2, since the difference between the experimental and calculated diffraction profiles in model A exceeded those in models B and C, better refinements were achieved using hydrogenation models B or C rather than non-hydrogenation model A in the diffraction data. For #085702, the weighted reliability factor (*R*_wp_) of the Rietveld refinement of models A–C were 6.130%, 5.794%, and 5.691%, respectively, and the reduced chi-square (*S*^2^) values were 1.290, 1.154, and 1.158, respectively. The hydrogen content (*x*) in γ-FeH_*x*_ calculated from *x* = *g*_H(O)_ + 2*g*_H(T)_ was 0.132(10) in model B and 0.154(15) with *g*_H(O)_ = 0.140(12) and *g*_H(T)_ = 0.007(4) in model C. This indicated that the hydrogen content in model B was consistent with that in model C within the fitting errors. In addition, density functional theory calculations reported by Antonov et al.^30^ pointed out that the energy of the T-site occupation significantly exceeded that of the O-site occupation. Thus, we concluded that model B was more appropriate than model C. The Rietveld refinement results for γ-FeH_*x*_ using model B are presented in Extended Data Table 2.

**Table 2:**
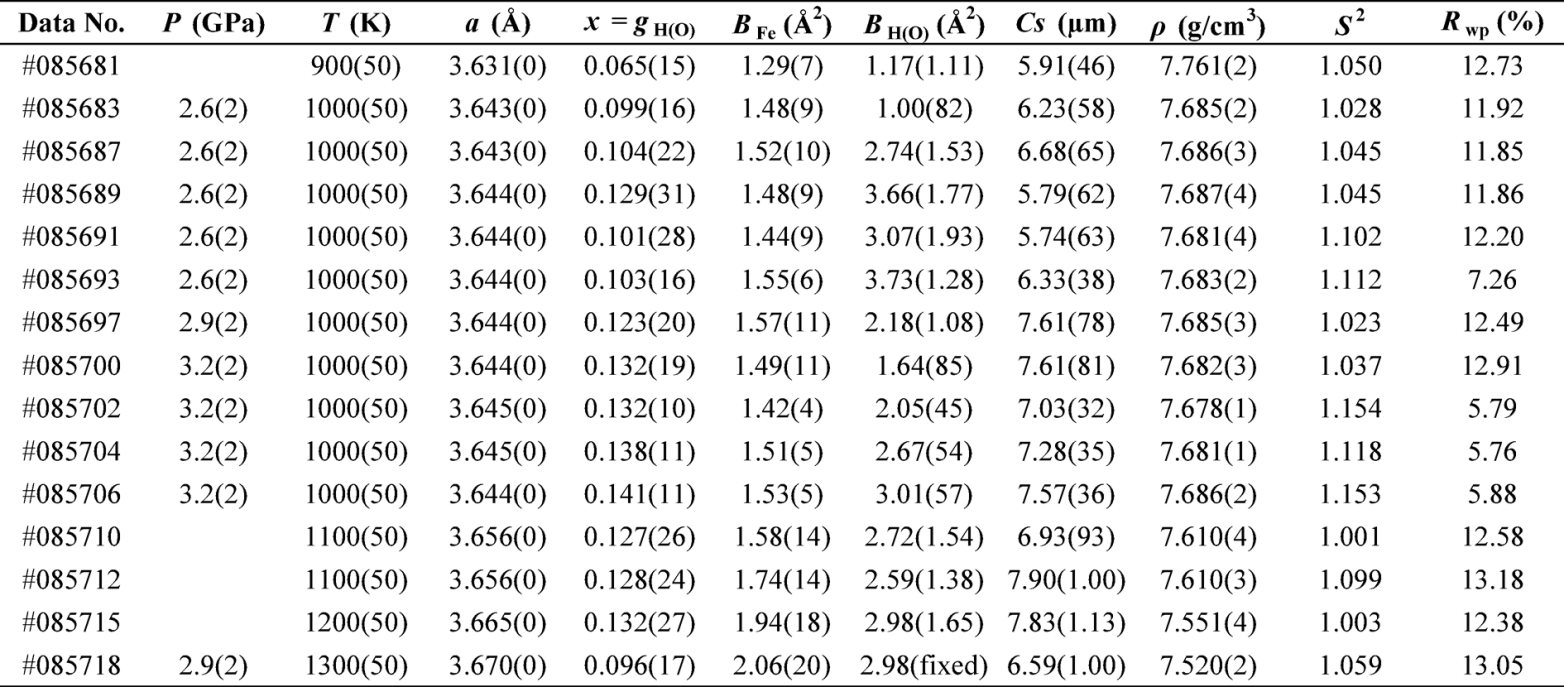
Rietveld refinement results for γ-FeH_x_.

## Results of neutron imaging experiments

**Fig. 3:**
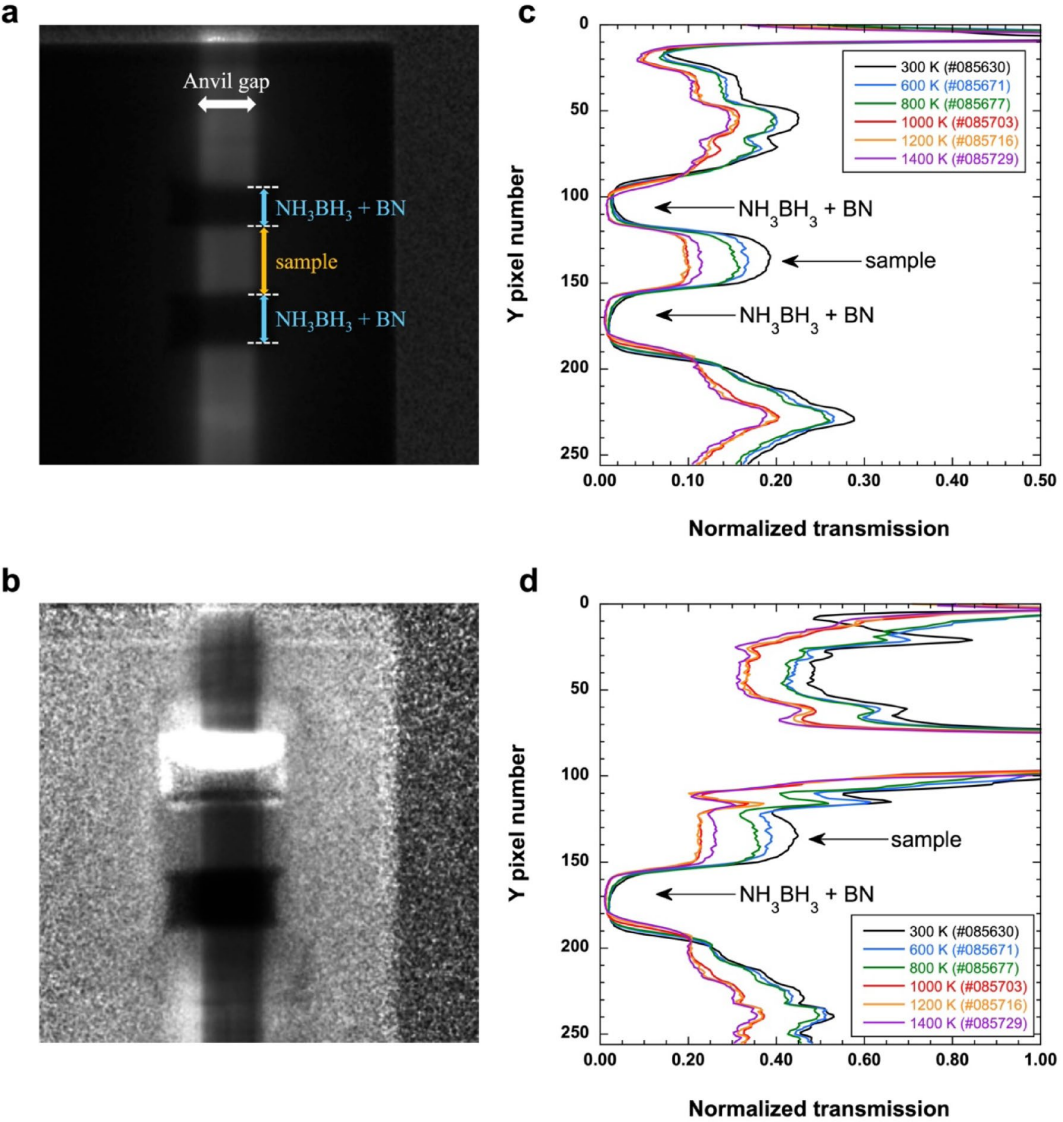
Results of neutron imaging experiments. Typical neutron radiography images (**a**) normalized by the proton number and direct beam image, as well as the (**b**) proton number and empty cell image. (**c**) and (**d**) Normalized neutron transmission profiles with the Y pixel number of (**a**) and (**b**), respectively.

Neutron imaging experiments were conducted alternately with the acquisition of neutron diffraction data. The experiments were performed using MiniPIX ver. 1.0 (ADVACAM s.r.o.), which provides an array of 256 × 256 pixels of a 55-µm pixel pitch (see “Methods”). The neutron radiography images of the sample were collected through an anvil gap (approximately 2 mm wide). Figure 3a and b show typical neutron radiography images obtained at 3.2 GPa and 1000 K, normalized by the proton number and direct beam image (Fig. 3a), as well as the proton number and empty cell image (Fig. 3b). Figure 3c and d show the normalized neutron transmission profiles along with the Y pixel number. The neutron transmission of the sample decreased during a phase transition from α-Fe to γ-FeH_*x*_. After melting, the neutron transmission slightly increased.

Based on the Beer–Lambert law, the linear absorption coefficient of the sample can be obtained from the sample length and the normalized transmission of the neutron radiography images divided by the proton number and empty cell image (see “Methods”). Assuming that the high-pressure cell was isotropically compressed, the sample lengths in the direction parallel to the neutron beam axis were equal to those obtained from the neutron radiography image. They were ~ 0.4 cm in all the neutron radiography images obtained at approximately 3 GPa (see “Methods”). The normalized neutron transmission was measured from the intensity of the center of the sample image normalized by the proton number and empty cell image. Extended Data Table 3 summarizes the results.

**Table 3:**
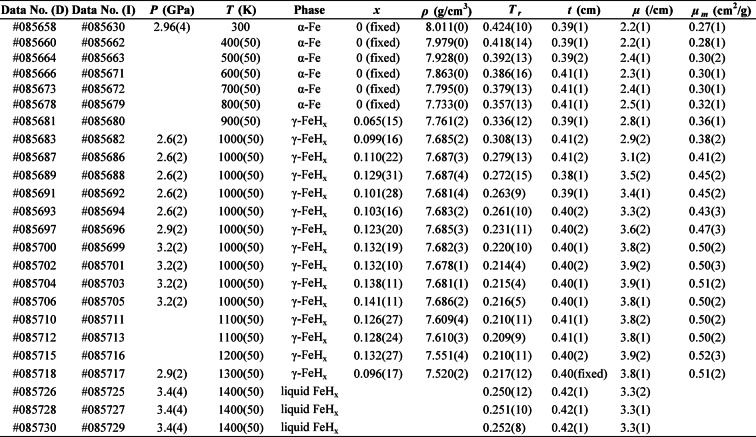
Summary of experimental conditions and results.

The values in parentheses represent the error of each parameter. The “fixed” in parentheses means the parameter was fixed. D, diffraction; I, imaging; *P*, pressure; *T*, temperature; *x*, hydrogen content; *ρ*, density; T_r_, normalized transmission; *t*, sample length; *µ*, linear absorption coefficient; µ_m_, mass absorption coefficient.

### Hydrogen content in liquid iron

**Fig. 4:**
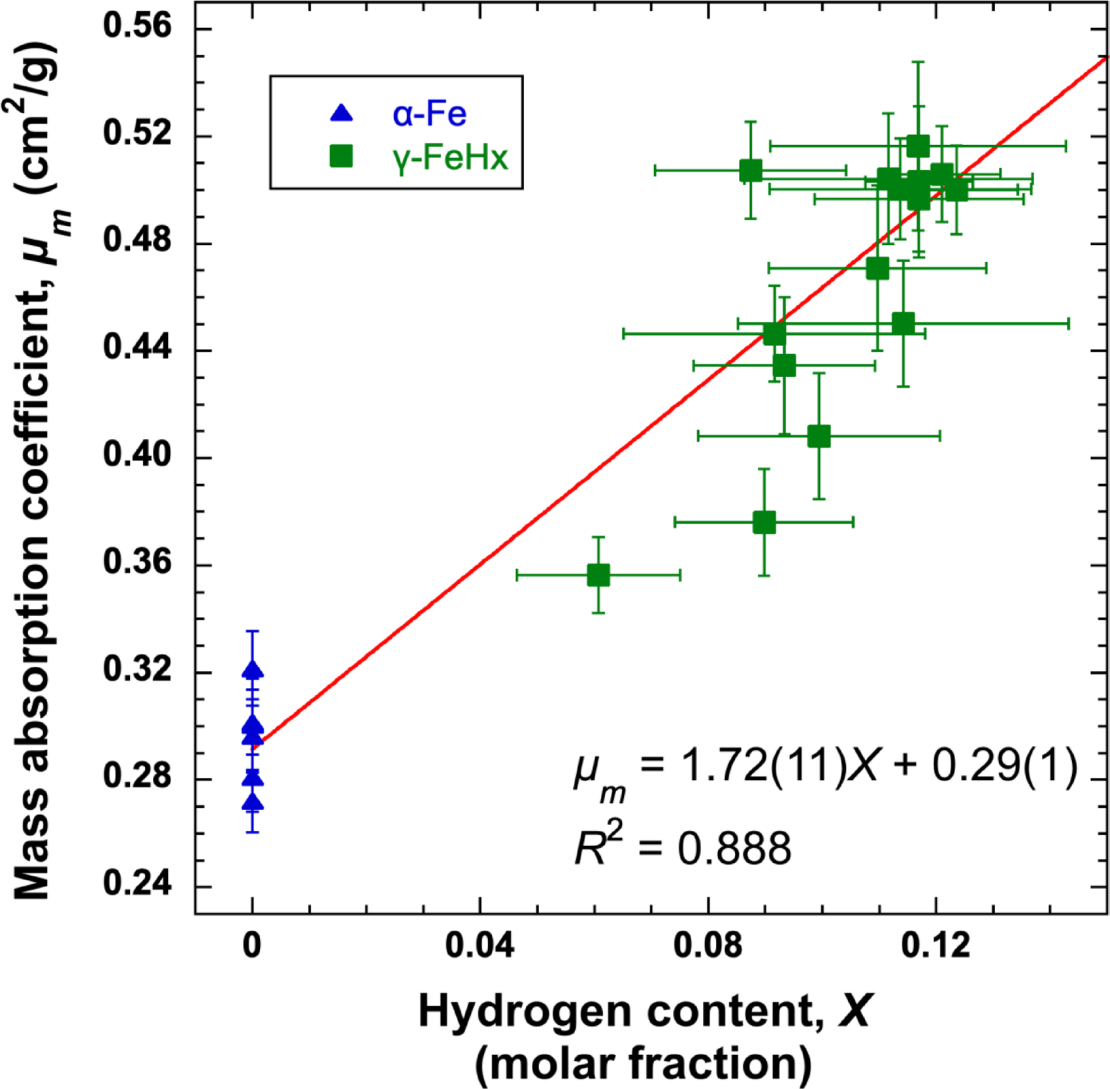
Mass absorption coefficient as a function of the hydrogen content. The red line represents the calibration curve fitting of the linear model with York’s method. The coefficient of determination (*R*^2^) suggests a good linear correlation between the hydrogen content and mass absorption coefficient.

To prevent the effect of the density (*ρ*) on the linear absorption coefficient (*µ*), the mass absorption coefficient (*µ*_*m*_), independent of the phase, pressure, and temperature, should be used for the calibration curve. The *µ*_*m*_ is defined as *µ*_*m*_ = *µ*/*ρ* and is shown in Extended Data Table 3. Figure 4 shows the *µ*_*m*_ as a function of *X*, the molar fraction of hydrogen, from the α-Fe and γ-FeH_*x*_ data. We calibrated the mass absorption coefficient with hydrogen molar fraction using a linear model. To test potential curvature, we also fitted a quadratic form. The t-test for the quadratic term yielded *p* = 0.18, indicating that the quadratic term is not statistically different from zero for our dataset. This linearity is further consistent with neutron imaging results on zirconium hydrides^31^, where *µ*_*m*_ scales approximately linearly with hydrogen content over comparable composition ranges. Assuming a linear correlation between the molar fraction of hydrogen and *µ*_*m*_, we created a calibration curve to determine the hydrogen content in liquid FeH_*x*_ from its mass absorption coefficient, as shown in the following equation:

1 $$\:\begin{array}{c}{\mu\:}_{m}=1.72\left(11\right)\times\:X+0.29\left(1\right).\end{array}$$

However, the *µ*_*m*_ of liquid FeH_*x*_ cannot be calculated because the density of liquid FeH_*x*_ is unknown. Thus, we estimated the density as a function of the hydrogen content using the equation of state (EoS) for liquid FeH_*x*_ determined by Stoutenburg et al.^32^. They developed the pressure, volume, temperature, and composition (*PVT*-*X*) EoS for liquid FeH_*x*_ through ab initio molecular dynamics (MD) simulations. Using the *PVT*-*X* EoS, the molar volume of liquid FeH_*x*_ was calculated as a function of the molar fraction of hydrogen at 3.4 GPa and 1400 K. Thus, the density of liquid FeH_*x*_ was given as a function of *X*, and the *µ*_*m*_ of liquid FeH_*x*_ was calculated as a function of *X* using the linear absorption coefficient. Combining the *µ*_*m*_ of liquid FeH_*x*_ with the calibration curve (Eq. 1), the hydrogen content was determined to be *X* = 0.086(12) in the molar fraction or *x* = 0.094(14) at a H/Fe ratio, corresponding to 0.17(3) wt% H at 3.4 GPa and 1400 K. In estimating *X*, we propagated uncertainties from the linear absorption coefficients in liquid FeH_*x*_ (*µ*), the *X*-*µ*_*m*_ calibration coefficients (*a*, *b*), and the EoS parameters.

Under low pressures (< 1 MPa), the solubility of hydrogen in metals in equilibrium with gaseous hydrogen is well described by Sieverts’ law as long as hydrogen can be regarded as an ideal gas^31^. However, the hydrogen content in iron is enhanced by an order of magnitude over the prediction from Sieverts’ law under higher pressures in the GPa range^10,33^. Although deviations from Sieverts’ law are expected to occur at several gigapascals^10,33^, the hydrogen solubility model based on the modified Sieverts’ law can be used to estimate the hydrogen content of liquid FeH_*x*_ under high pressures.

Under low pressures, Sieverts’ law successfully describes the dissolution of hydrogen in pure liquid iron^33,34^ as follows:

2 $$\:\begin{array}{c}\frac{1}{2}{\mathrm{H}}_{2}\left(\mathrm{g}\mathrm{a}\mathrm{s}\right)\iff\:{\mathrm{H}}\:\left(\mathrm{i}\text{n }\:\mathrm{l}\mathrm{i}\mathrm{q}\mathrm{u}\mathrm{i}\text{d }\:\mathrm{p}\mathrm{u}\mathrm{r}\text{e }\:\mathrm{i}\mathrm{r}\mathrm{o}\mathrm{n}\right).\:\end{array}$$

Following Sieverts’ law, the amount of hydrogen dissolved in liquid iron is proportional to the square root of the hydrogen partial pressure:

3 $$\:\begin{array}{c}\left[\mathrm{H}\right]={K}_{\mathrm{S}}\times\:\sqrt{\frac{{P}_{{\mathrm{H}}_{2}}}{{P}_{{\mathrm{H}}_{2}}^{\circ\:}}},\:\end{array}$$

where [H] is the hydrogen solubility in liquid iron in wt%; and *K*_s_ is Sieverts’ constant, which is the hydrogen solubility under a hydrogen partial pressure of 1 atm. $\:{P}_{{\mathrm{H}}_{2}}$ is the hydrogen partial pressure, and $\:{P}_{{\mathrm{H}}_{2}}^{\circ\:}$ is the 1-atm pressure.

Under a pressure of 1 atm, the solubility of hydrogen in liquid iron is determined via various methods (Table II in a study by Boorstein and Pehlke^34^. The enthalpy of the solution was positive^35^, indicating that the solution was dissolved endothermically. The temperature dependence of the hydrogen solubility can be fit with an exponential relationship:

4 $$\:\begin{array}{c}\left[\mathrm{H}\right]=B\mathrm{exp}\left(-\frac{C}{T}\right),\:\end{array}$$

with *B* and *C* as the coefficients. Zhang et al.^36^ compiled data on the hydrogen solubility in pure liquid iron under a pressure of 1 atm. Using their data, *B* and *C* were determined to be 2.16 × 10^− 2^ and 4010, respectively.

To model the hydrogen solubility in liquid iron under high pressure at a high temperature, we combined Eqs. (3) and (4) as follows:

5 $$\:\begin{array}{c}\left[\mathrm{H}\right]\left(P,\:T\right)=A\times\:\sqrt{\frac{{P}_{{\mathrm{H}}_{2}}}{{P}_{{\mathrm{H}}_{2}}^{\circ\:}}}+B\mathrm{exp}\left(-\frac{C}{T}\right).\end{array}$$

Because no systematic data exist for the temperature dependence of hydrogen solubility in liquid iron under high pressure, and structural studies have shown that the structure of liquid iron changes little with pressure^37^, we assumed that the temperature dependence of solubility is similar to that at ambient pressure. The experimental data were fitted in with Eq. (5), and the solubility coefficient *A* was determined to be 9.19 × 10^− 4^. Incidentally, the hydrogen content under conditions of 1 atm and 1873 K was calculated to be 34.6 ppm H using this model, roughly consistent with the measured value of 24.7 ppm H (27.7 cc of hydrogen per 100 g of iron) at 1 atm and 1873 K^34^. Extended Data Fig. 5 shows the model as a contour plot of the hydrogen content as a function of pressure and temperature.

**Fig. 5:**
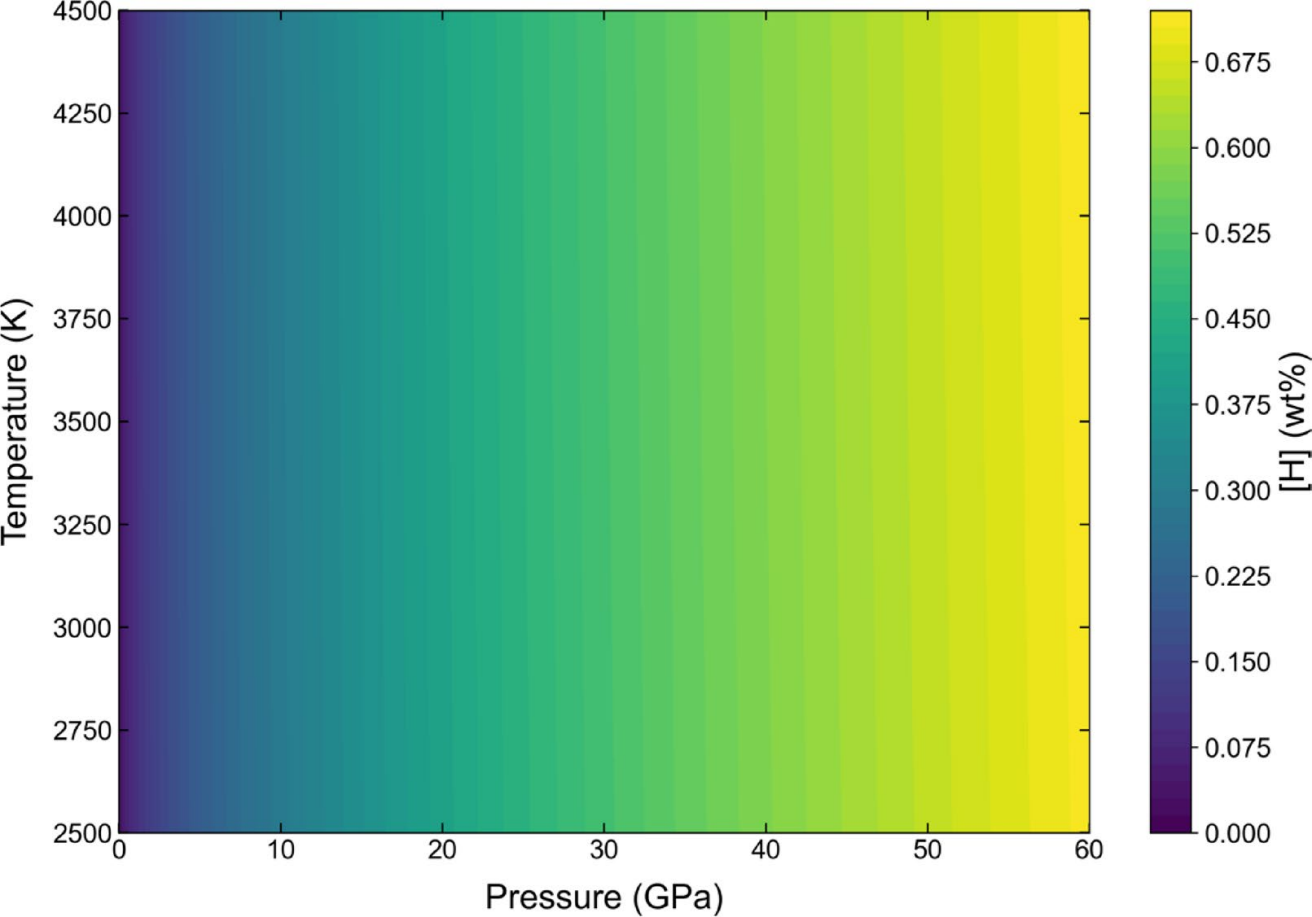
Contour plot of the H content as a function of pressure and temperature. The H content was calculated using Eq. (5) and was more dependent on pressure than on temperature.

### Implications for the Earth’s core

During accretion, the gravitational potential energy of impacting planetesimals supplies sufficient energy to form a magma ocean. According to the single-stage core formation model^38–40^, droplets of liquid metals descend through the magma ocean, equilibrating with the surrounding silicate melt, and the liquid metals pond at the base of the magma ocean. Thereafter, liquid metals form large diapirs, which rapidly fall to the growing core without further equilibration with the surrounding silicate. During core segregation, it is suggested that the hydrogen dissolved in the magma ocean is separated from silicate melt into liquid iron because hydrogen is strongly siderophile under core-forming conditions^5–7,24^. Recent studies show that hydrogen probably dissolved in the magma ocean via in-gassing from the solar nebular gas when the proto-Earth reached sufficient mass to capture the hydrogen-rich primary atmosphere from the hydrogen-dominated solar nebula^41,42^. Under such hydrogen-rich conditions, liquid Fe droplets can contain ~ 0.17 wt% H at a depth corresponding to ~ 3 GPa, as observed in this study. Thereafter, the liquid iron droplets containing hydrogen segregate from the silicate magma ocean and form the Earth’s metallic core. Our results provide experimental constraints on the possible incorporation of hydrogen into metallic iron under core-forming conditions, supporting the hypothesis that hydrogen may be one of the light elements in the core.

The equilibrium condition between the magma ocean and liquid metals has been refined by the metal-silicate partitioning of certain siderophile elements^38–40^. We estimated the hydrogen content in the Earth’s core using our hydrogen solubility model. While determining the hydrogen content of liquid iron in equilibrium with silicate melt at the base of a magma ocean requires incorporation of the hydrogen/water activities in silicate melt and the oxygen fugacity, our solubility model should be regarded as providing an upper bound on the hydrogen content that could dissolve into core-forming metal. As shown in Extended Data Table 4, the hydrogen content in liquid iron at the base of the magma ocean was calculated using the hydrogen content as a function of pressure and temperature (Eq. 5 and Extended Data Fig. 5). Assuming hydrogen as the only light element in the core and extremely hydrogen rich conditions, the core-forming liquid iron is presumed to contain 0.59–0.70 wt% H at the base of the magma ocean. In addition, hydrogen probably dissolves more in liquid iron than in solid iron, as observed in experiments^18,19,24^ and predicted via MD simulations^43,44^. Yuan and Steinle-Neumann^44^ determined the hydrogen partition coefficient (*D*^s/l^, the ratio of mass fractions of hydrogen in solid and liquid iron) to be within the range of 0.50(3)–0.62(3) under inner core boundary (ICB) conditions. Combining the *D*^s/l^ with the hydrogen budget of the bulk core in the range of 0.59–0.70 wt% H estimated in this study, the outer core of 95% total core mass sequestered 0.60–0.72 wt% H, and the inner core had been slightly depleted with 0.30–0.44 wt% H. This estimate indicates that the bulk core contains 72–87 times the mass of hydrogen in the ocean (ocean mass = 1.41 × 10^21^ kg^45^), with 70–85 and 1.9–2.7 times the mass of hydrogen in the ocean for the outer and inner cores, respectively. This is consistent with the estimation of Stoutenburg et al.^32^, who calculated the hydrogen content that reproduces the density of the outer core by comparing the preliminary reference Earth model (PREM)^46^ and estimated the ranges of 0.66–1.10 and 0.49–0.72 wt% H at the core–mantle boundary (CMB) and ICB, respectively. Additionally, the present results are roughly consistent with those obtained by Thompson et al.^16^, who estimated 0.8–1.3 and 0.2–0.6 wt% H at the CMB with *T*_CMB_ = 4000 K and *T*_ICB_ = 5500 K, respectively, by comparing the PREM with the EoS of pure iron and γ-FeH_*x*_. Through first-principles MD simulations, Umemoto and Hirose^47^ observed that the density and compressional wave velocity of liquid iron containing ~ 1 wt% H matched the PREM at the outer core. Our results indicate that hydrogen could account for a major portion of the density deficit of the outer core if it is the sole light element; however, additional light elements are likely necessary to explain the full density deficit of the outer core even under extremely hydrogen rich conditions.

**Table 4:**
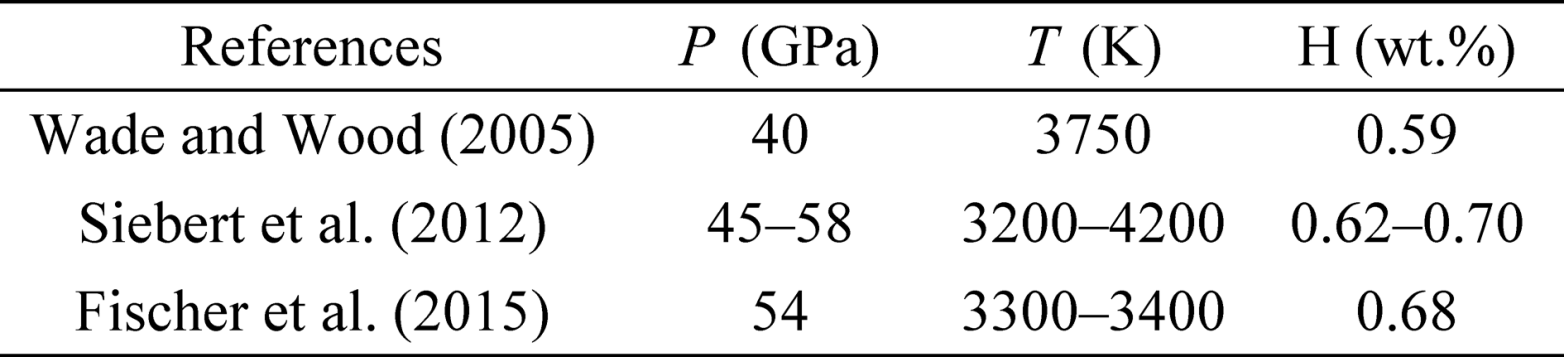
Estimated hydrogen content at the base of the magma ocean.

## Methods

### High-pressure neutron experiments

High-pressure and high-temperature neutron diffraction and imaging experiments were conducted at the PLANET beamline (BL11) at the Materials and Life Science Experimental Facility (MLF) in J-PARC. The high pressure and temperature were generated with a six-axis multi-anvil apparatus (ATSUHIME) installed in the beamline using a 6–6 cell assembly^25,26^. Tungsten carbide anvils (truncated edge length = 10 mm) were used as second-stage anvils. A guide frame made of an Al alloy with slits for incident, transmitted, and diffracted neutrons was used for bundling six second-stage anvils and a high-pressure cell. The assembled cell was covered with epoxy glass cloth–laminated sheets (0.5-mm thick) to prevent the leakage of the radioactive cell assembly. The high-pressure cell assembly (Extended Data Fig. 6) was designed based on the conventional design used by a previous neutron diffraction study^20^. A sample of pure iron powder (Mitsuwa Chemicals Co., Ltd.) was pressed into a pellet and placed in the center of a hydrogen-sealing capsule made of NaCl with internal hydrogen sources of NH_3_BH_3_ pellets (Tokyo Chemical Industry Co., Ltd.) on the top and bottom sides of the sample. When the NH_3_BH_3_ was heated beyond 573 K under any pressure from 0.6 to 9 GPa, it irreversibly decomposed to H_2_, and chemically inert amorphous BN does not react with hydrogen^48^. The initial molar ratio H/Fe was set to ~ 2 to ensure the hydrogen-saturated condition. A graphite cylinder and disks were used as a furnace, and the electrodes were Au foils (50-µm thick). The NaCl capsule was inserted into a cylindrical graphite heater and embedded in a pressure-transmitting medium made of ZrO_2_ (17-mm edge cube), with high transparency for neutrons. The generated pressure was determined from the lattice parameters of the NaCl capsule using the EoS for NaCl^49^. The experimental temperature was estimated from the electric power based on a power–temperature calibration curve. The uncertainty of the temperature was estimated at ± 50 K.

**Fig. 6:**
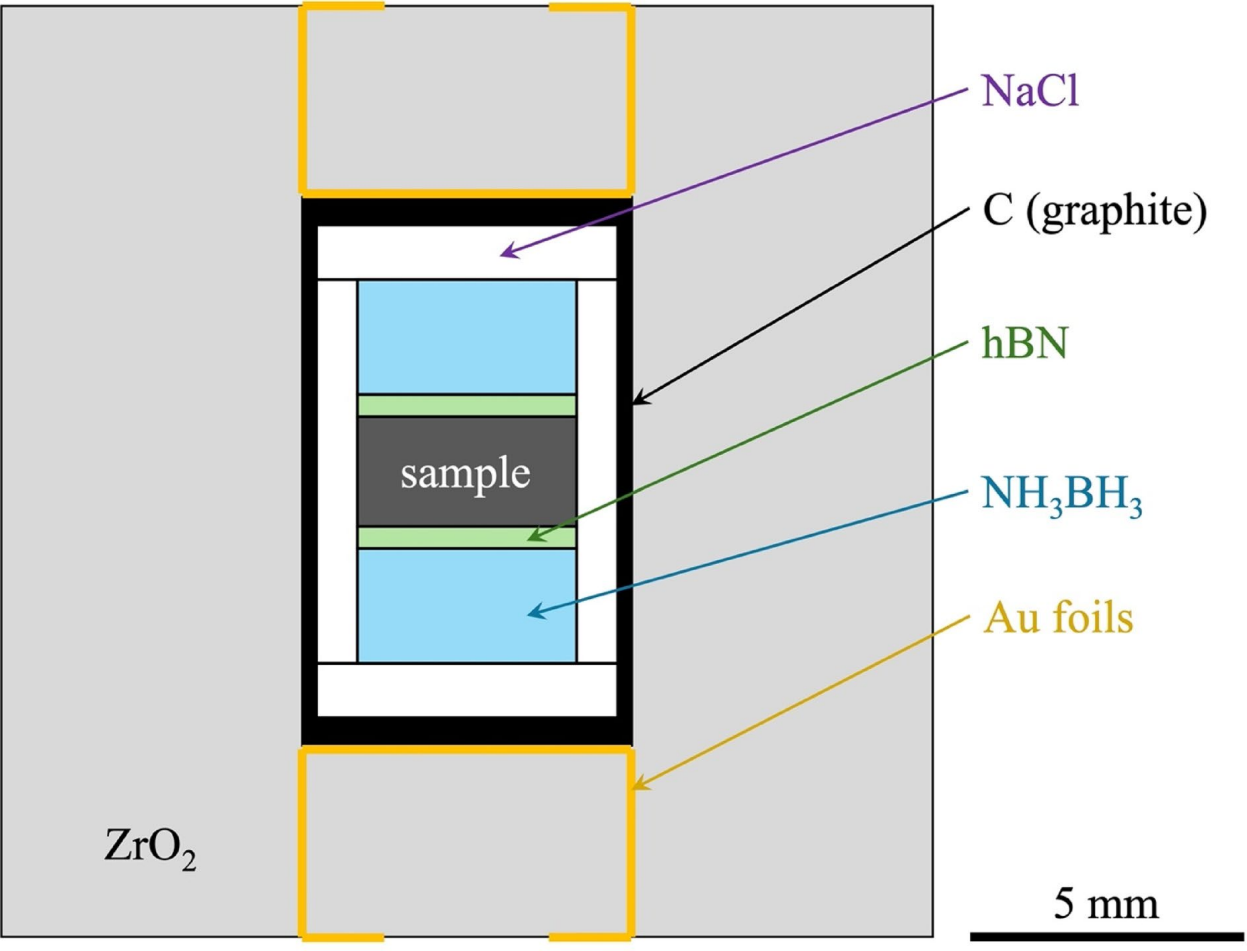
Experimental setup. Schematic illustration of the high-pressure cell assembly.

### Neutron diffraction experiments and Rietveld refinement

Neutron diffraction profiles, accumulated for 10–60 min, were recorded every 100 K up to 1400 K at approximately 3 GPa. For data analysis, the diffraction intensity of the sample was corrected using the data for a vanadium pellet and an empty cell based on a procedure described by Hattori et al.^26^. The corrected diffraction data were refined via the Rietveld method^50^ with the Z-Rietveld software^51,52^ to precisely determine the hydrogen contents and structural parameters. The crystal symmetry of γ-FeH_*x*_ is cubic (*Fm*_3*m*, *Z* = 4), and the atomic sites of Fe are 4*a* (0, 0, 0). The O-site of γ-FeH_*x*_ are 4*b* (1/2, 1/2, 1/2), and the T-site of γ-FeH_*x*_ are 8*c* (1/4, 1/4, 1/4). For refinement, extinction correction was conducted using the crystallite size *C*_*S*_ as a variable parameter. In addition, considering the harmonic oscillation approximation for H atoms, we applied a constraint on the atomic displacement parameters for H atoms occupying the O- and T-sites using the following equation:

6 $$\:\begin{array}{*{20}{c}} {\frac{{{B_{\mathrm{H(O)}}}}}{{{B_{\mathrm{H(T)}}}}} = \frac{{{{\left\langle {\hbar \:\omega } \right\rangle }_{\mathrm{H(T)}}}\coth \left( {\frac{{{{\left\langle {\hbar \:\omega } \right\rangle }_{\mathrm{H(O)}}}}}{{2{k_B}T}}} \right)}}{{{{\left\langle {\hbar \:\omega } \right\rangle }_{\mathrm{H(O)}}}\coth \left( {\frac{{{{\left\langle {\hbar \:\omega } \right\rangle }_{\mathrm{H(T)}}}}}{{2{k_B}T}}} \right)}},\:} \end{array}$$

where $\:{B}_{\mathrm{H}\left(\mathrm{O}\right)}$ and $\:{B}_{\mathrm{H}\left(\mathrm{T}\right)}$ are the atomic displacement parameters for H atoms at the O- and T-sites, respectively, *k*_B_ is Boltzmann constant, and *T* is temperature. ${\left\langle {\hbar \:\omega } \right\rangle _{\mathrm{H(O)}}}$ and ${\left\langle {\:\hbar \:\omega } \right\rangle _{\mathrm{H(T)}}}$ are the mean energies of optical hydrogen vibrations for the O- and T-sites, respectively; the values reported by Antonov et al.^30^ were used. Thus, the structural parameters, site occupancies of H at the O- and T-sites (*g*_H(O)_ and *g*_H(T)_), *C*_*S*_, and the isotropic atomic displacement parameters ($\:{B}_{\mathrm{F}\mathrm{e}}$, $\:{B}_{\mathrm{H}\left(\mathrm{O}\right)}$, and $\:{B}_{\mathrm{H}\left(\mathrm{T}\right)}$), were refined.

### Neutron imaging

Neutron radiography images were collected alternately with the acquisition of the neutron diffraction data. A miniaturized radiation camera (MiniPIX, ver. 1.0, ADVACAM s.r.o.) was used for high-pressure neutron imaging experiments. The detector provides an array of 256 × 256 pixels of a 55-µm pixel pitch, resulting in a detection area of 14.08 × 14.08 mm = 1.98 cm^2^(ref^53^.). Each pixel of the MiniPIX detector can be controlled to run in one of four operating modes. Here, we selected the Time-over-Threshold mode, in which the counter is continuously incremented as long as the signal is over the threshold. The exposure time was set to 1 s, and measured frames were summed into one frame. Prior to the experiment, a direct beam (flat field) image was taken to record the distribution of the beam without the sample, and the empty cell image was collected separately after the experiments. The ImageJ software was used for image analysis. The collected images were normalized to proton numbers for each image and divided by the direct beam image for flat-field correction.

The measured intensity of the transmitted neutron, $\:I$, was calculated, following the Beer–Lambert law as follows:

7 $$\:\begin{array}{c}I={I}_{0}{e}^{-\mu\:t},\:\end{array}$$

where $\:{I}_{0}$ is the direct beam intensity, $\:\mu\:$ is the linear attenuation coefficient (cm^[–1^, and $\:t$ is the sample length (cm). The intensity of the transmitted neutron through the high-pressure cell, which is affected by the absorption in the sample, capsule, and environment, can be denoted by

8 $$\:\begin{array}{c}{I}_{s}={I}_{0}\mathrm{exp}\left\{\left(-{\mu\:}_{s}{t}_{s}\right)+\left(-{\mu\:}_{c}{t}_{c}\right)+\left(-{\mu\:}_{e}{t}_{e}\right)\right\},\:\end{array}$$

where the subscripts of *s*, *c*, and *e* represent the sample, capsule, and environment, respectively. Similarly, the intensity of the transmitted neutron through the empty cell (without the sample) is

9 $$\:\begin{array}{c}{I}_{\mathrm{empty}}={I}_{0}\mathrm{exp}\left\{\left(-{\mu\:}_{c}{t}_{c}\right)+\left(-{\mu\:}_{e}{t}_{e}\right)\right\}.\:\end{array}$$

Thus, the contribution of absorption in the sample can be obtained using Eqs. (8) and (9),

10 $$\:\begin{array}{c}\frac{{I}_{s}}{{I}_{\mathrm{empty}}}=\mathrm{exp}\left(-{\mu\:}_{s}{t}_{s}\right).\end{array}$$

The linear absorption coefficient can be obtained from the sample length and the intensity of the neutron radiography image divided by that of the empty cell. Assuming that the high-pressure cell was isotropically compressed, the sample length ($\:{t}_{s}$) in the direction parallel to the neutron beam axis was equal to the sample length obtained from the neutron radiography image.

The sample length was determined from the neutron radiography images normalized by the proton number and direct beam image. Extended Data Fig. 7 shows the methodology and an example of the estimation of the sample length. Normalized neutron transmission gradually changes at the boundary between the sample and NaCl capsule, and the sample area transmission must be lower than that of the NaCl capsule. Edge spread function (ESF) profiles were obtained along with the X pixel number, and line spread function (LSF) profiles were calculated by differentiating the ESF. Thereafter, the centers of the LSF peaks, as the left and right ends of the sample, were determined via Gaussian fitting for the LSF profiles. The sample lengths were estimated from the differences between the right and left ends of the sample. Here, the measured sample lengths were ~ 0.4 cm in all the neutron radiography images taken at approximately 3 GPa.

**Fig. 7:**
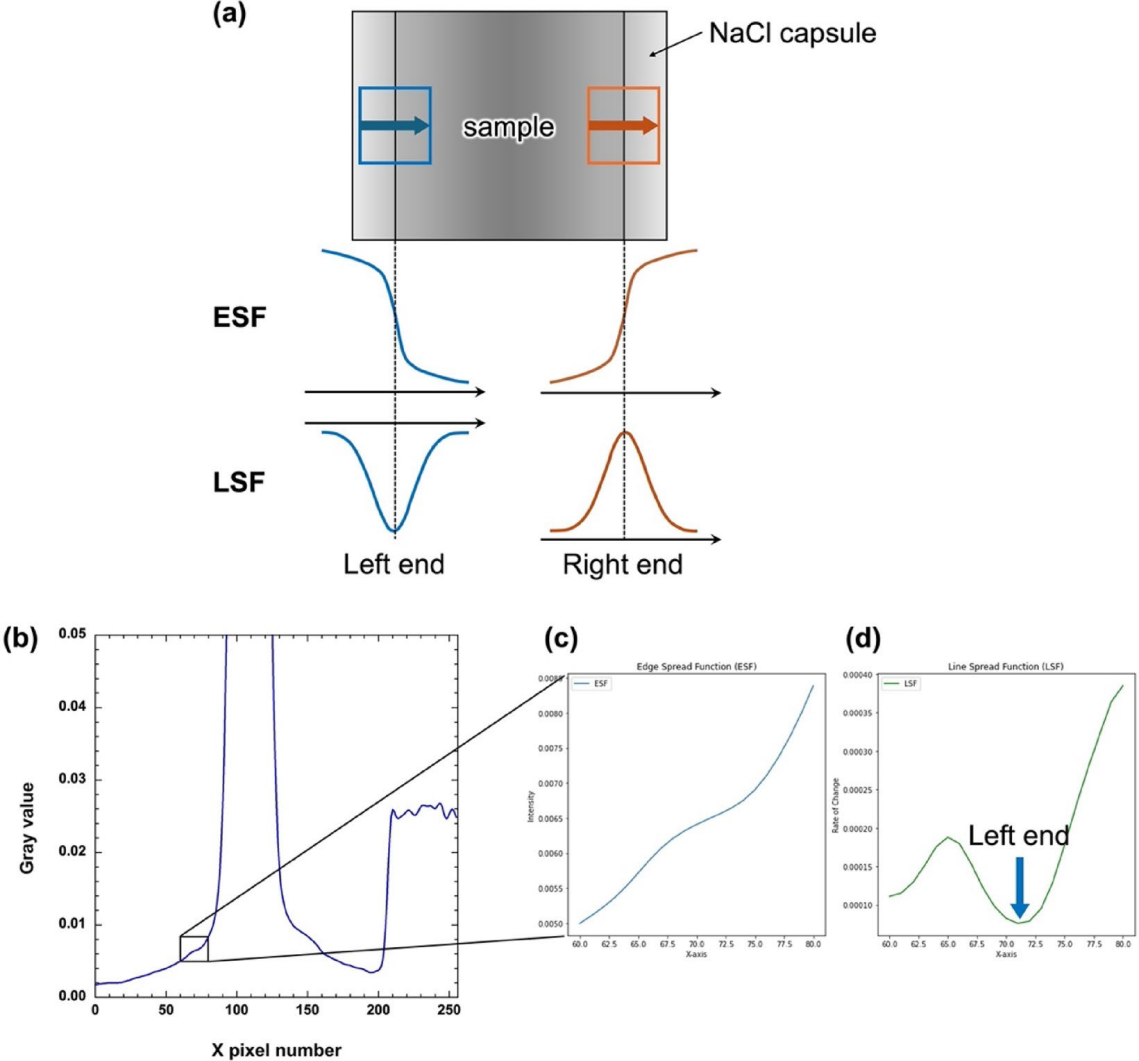
Schematic illustration and example of the determination of the sample length. (**a**) Schematic illustration for the detection of the left and right ends of the sample. (**b**) Example of edge spread function (ESF) profiles. (**c**) Enlarged profiles of the area surrounded by a black square in (b). (**d**) Line spread function (LSF) profiles of (c). The blue arrow indicates the left end of the sample.

## Data Availability

The datasets used and analysed during the current study are available from the corresponding author on reasonable request.
